# Natural Orifice Transesophageal Endoscopic Surgery: State of the Art

**DOI:** 10.1155/2012/896952

**Published:** 2012-04-09

**Authors:** João Moreira-Pinto, Aníbal Ferreira, Carla Rolanda, Jorge Correia-Pinto

**Affiliations:** ^1^Life and Health Sciences Research Institute (ICVS), School of Health Sciences, University of Minho, 4709-057 Braga, Portugal; ^2^ICVS/3B's, PT Government Associate Laboratory, 4709-057 Braga/Guimarães, Portugal; ^3^Department of Pediatric Surgery, Centro Hospitalar do Porto, 4099-001 Porto, Portugal; ^4^Department of Gastroenterology, Hospital de Braga, 4710-243 Braga, Portugal; ^5^Department of Pediatric Surgery, Hospital de Braga, 4710-243 Braga, Portugal

## Abstract

The main goal of Natural Orifice Transluminal Endoscopic Surgery (NOTES) is performing surgery avoiding skin incisions. Theoretical advantages of NOTES include decreased postoperative pain, reduction/elimination of general anesthesia, improved cosmetic outcomes, elimination of skin incision-related complications such as wound infections and hernias, and increased overall patient satisfaction. Although various forms of port creation to accomplish thoracic NOTES procedures have been proposed, transesophageal NOTES has been shown to be the most reliable one. The evolution of endoscopic submucosal transesophageal access resulted in the development of per-oral endoscopic myotomy (POEM), which had a fast transition to clinical practice. The authors present a review of the current state of the art concerning transesophageal NOTES, looking at its potential for diagnostic and therapeutic interventions as well as the hurdles yet to be overcome.

## 1. Introduction

Natural Orifice Transluminal Endoscopic Surgery (NOTES) is the name given to novel endoscopic interventions on internal organs performed through natural orifices. In this new approach, endoscopes enter the abdominal and thoracic cavities via any single or combination of natural orifices—mouth, urethra, vagina, and anus [1]. In fact, NOTES dates back to 1940s, when Decker performed the first culdoscopy using an endoscope passed through the rectouterine pouch to view pelvic organs and perform sterilization procedures [2]. These procedures were superseded by noninvasive ultrasound imaging for diagnostic purposes and laparoscopy for surgical purposes. Later, NOTES was to be reborn when Rao and Reddy presented the video of the first transgastric appendectomy at the 2004 Annual Conference of the Society of Gastrointestinal Endoscopy of India [3]. In a severely burnt patient, whose skin they could not incise, they used a therapeutic flexible gastroscope to reach his stomach. Then, they performed an inside-out gastrostomy and pushed the gastroscope through the gastric wall into the abdominal cavity. They looked for the appendix and performed the first ever transgastric appendectomy.

The first description of transgastric peritoneoscopy in porcine model published in paper was by Kallo et al. in 2004 [4]. Soon, other natural orifices were presented as good access points for NOTES. Pai et al. published transcolonic peritoneoscopy followed by a series of transcolonic procedures [5]. The access from below gives a good, direct view of the upper abdominal cavity. Having this in mind, Lima et al. presented transvesical endoscopic peritoneoscopy [6]. To accomplish NOTES procedures in the thorax and the mediastinum, Sumiyama et al. proposed a transesophageal access [7]. Transvesical-transdiaphragmatic [8], transgastric-transdiaphragmatic [9], and transtracheal [10] access have been suggested too. Even though, the transesophageal has been preferred as a direct entry to the thorax and permited several procedures in porcine model (Table 1) [11–19].

The main goal of NOTES is to avoid skin incisions and its associated complications, such as wound infections and hernias. Theoretical advantages of NOTES include reduction in hospital stay, faster return to bowel function, decreased post-operative pain, reduction/elimination of general anesthesia, performance of procedures in an outpatient or even office setting, possibly cost reduction, improved cosmetic outcomes, and increased overall patient satisfaction [1].

## 2. Transesophageal Approach

When Sumyiama et al. presented transesophageal access to the thorax and mediastinum, they used submucosal endoscopy with mucosal flap (SEMF) [7]. The authors injected saline into the esophageal submucosal layer creating a bleb and high-pressure carbon dioxide was used to perform a submucosal dissection. A biliary retrieval balloon was then inserted into the submucosal layer and was distended to enlarge the mucosal hole and create a 10 cm long submucosa tunnel. Subsequently, they used an endoscopic mucosal resection (EMR) cap (Olympus, Tokyo, Japan) to create a defect in the muscularis propria and the mediastinum was entered. The key of the method is the overlying mucosa which serves as a sealant flap minimizing the risk of soiling a body cavity with lumenal contents and the ease by which the entry point into the submucosal working space can be closed [20].

**Figure 1:**
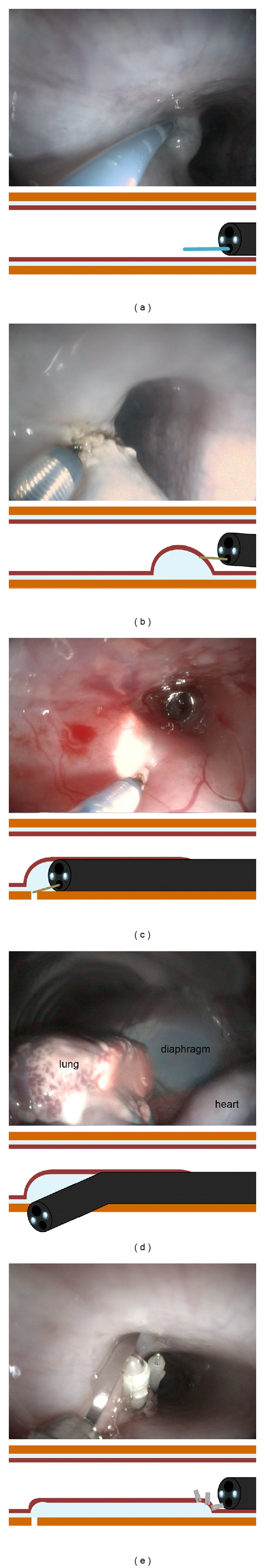
Transesophageal submucosal endoscopy with mucosal flap (SEMF) in a porcine model. (a) Saline is injected into the submucosal layer of the esophagus. (b) The mucosa of the bleb is incised using a needle knife. (c) A 10 cm tunnel is created using air and blunt dissection. The muscularis propria is incised at the distal end of the esophageal submucosal tunnel. (d) The endoscope is passed through the esophagotomy and the thoracic cavity is inspected. (e) Esophagotomy closure is achieved by mucosal flap adhesion. The mucosal defect is sutured using endoclips.

Several modifications have been described to SEMF (Figure 1). Mucosa can be incised using either needle knife, a prototype flexible CO_2_ laser fiber (OmniGuide Inc., Cambridge, MA, USA), or a Duette Multiband mucosectomy device (Cook Medical, Winston-Salem, NC, USA) [12]. Besides biliary retrieval balloons, the creation of the submucosal tunnel has been achieved with air and blunt dissection using snare tips, closed forceps, EMR caps [12–15]. Division of the muscular layer has been described using needle knife, although the aspiration method of the EMR cap may reduce the risk of injury to any adjacent mediastinal structure [13]. The SEMF procedure has also been applied in the stomach to safely perform NOTES in the abdominal cavity [21]. 

 According to *von Renteln *et al. working with the endoscope through a dissection tunnel limits endoscope movements and degrees of freedom, and major procedures tend to stretch open the submucosal tunnel resulting in a major defect or laceration [22]. On the other hand, Moyer et al. tested durability of submucosal endoscopic tunnel in the stomach and concluded that it tolerates the mechanical forces of peroral transgastric procedures provided that the organ resected is small to moderate in size (<8 × 3 cm) [23].

With or without submucosal tunneling, transesophageal approach to the thoracic cavity is highly risky because of possible mechanical abrasion and trauma of surrounding structures [13, 22]. For that, Fritscher-Ravens et al. proposed endosonographically EUS-assisted transesophageal access. In a comparative study of NOTES alone against EUS-assisted NOTES procedures, the authors found that the last was superior in gaining access, identifying structures, and therefore avoiding major complications [24].

A different alternative was presented by Rolanda et al. single transthoracic trocar assistance for transesophageal NOTES [18]. As most thoracic procedures imply some time of postoperative tube drainage, a 12 mm incision was made in the thoracic wall and a 10 mm trocar was inserted before esophagotomy was performed. Using a 10 mm thoracoscope with a 5 mm working channel (Karl Storz, Tuttlingen, Germany) inserted through the transthoracic trocar, transesophageal port was safety created with thoracoscopic visual control. Moreover, other well-known problems of NOTES, such as tissue manipulation, suturing, and anastomosis establishment, were overlapped, because triangulation and countertraction were achieved using flexible instruments inserted through the gastroscope and rigid instruments inserted through the thoracoscope. Therefore, transesophageal NOTES with the assistance of a single transthoracic trocar can be used for highly complex thoracic procedures.

Recently, our group has presented transesophageal pulmonary lobectomy with survival assessment in porcine model, using this single transthoracic port assistance [19]. Besides using flexible instruments inserted through the gastroscope, we introduced several rigid instruments through an oroesophageal overtube: endstaplers (EndoPath, Ethicon Endo-Surgery, Cincinnati, OH, USA), SILS-Stich (SILS stitch, Covidien, Mansfield, MA, USA), and knot-pusher. Coordinating the movement of a rigid instruments through the mouth with the image provided by the thoracoscope made ligation of the right upper bronchus and its vessels possible and reliable. The 12 mm thoracic incision was crucial for acute air and liquid drainage. All the four animals in the survival group subsisted for 15 days [19].

Transesophageal NOTES with the assistance of a single transthoracic trocar might be the key to incisionless cardiac procedures. Our group has performed left atrial appendage (LAA) ligation in 4 acute and 6 survival porcine models (unpublished results). The instruments entering both through the gastroscope and the thoracoscope made triangulation very similar to the one experienced on exclusive thoracoscopic approach. The flexible endoscope had a good access to all aspects of the heart—using direct position to reach the base of the heart and retroflexion for its apex. Moreover, flexible gastroscope was useful to show some parts of the thoracic cavity that could not be visualized with the 0° optic of the operative thoracoscope, namely, lateral thoracic wall and the entire diaphragm. With exception of the one acute experiment which was terminated because of LAA rupture, all the other animals were kept alive until the end of the experiment. No adverse event occurred during the survival period. Complete LAA ligation was verified on necropsy, as LAA was fibrotic with the nylon endo-loop in place.

The NOTES revolution permitted evolution of the different natural orifices approaches themselves. The performance of endoscopic submucosal transesophageal myotomy is a perfect example of this. Pasricha et al. used SEMF to perform peroral endoscopic myotomy (POEM) in an experimental setting [25]. Soon after this, Inoue et al. reported the first clinical experience of POEM for the treatment of achalasia [26]. In 17 consecutive patients, there were no intraoperative or postoperative complications, and the occasions of inadvertent entry into the cardiac mucosa (2 patients) and the exposure of mediastinal tissue (4 patients) were without incident. Although POEM might not be considered a true NOTES procedure because it does not divide all the layers of the esophagus, it does use readily available endoscopic equipment and techniques and directly competes with a laparoscopic procedure [27].

## 3. Esophagotomy Closure

When SEMF is used to create transesophageal access, esophagotomy closure is easy, as the overlying mucosa serves as a sealant flap. Most authors use endoclips to close the defect of the mucosa, but in the early studies the mucosa was left open with good clinical outcomes [7, 12–14]. Turner et al. published a study comparing esophageal submucosal tunnel closure with a stent versus no closure [28]. In this study, the unstented group achieved endoscopic and histologic evidence of complete reepithelialization and healing (100%) at the mucosectomy site compared with the stented group (20%, *P* = .048). So, it seems that the placement of a covered esophageal stent prejudices healing of the mucosectomy site.

When direct incision esophagotomy is performed, a full-thickness healing of the mucosal and muscular layer must be achieved. Fritscher-Raves et al. compared endoscopic clip-closure (ECC) versus endoscopic suturing (ECS) versus thoracoscopic (TC) repair of a 2–2.5 cm esophageal incision [29]. ECS was achieved using a prototype suturing system that deploys a metal anchor with a nonabsorbable polypropylene thread (T-bar) on each side of the esophageal defect (CR Bard, Murray Hill, NJ; Ethicon Endosurgery, Cincinnati, OH, USA). The two threads were joined together using a small cylindrical suture-locking device, approximating both sides of the incision. Three to 5 pairs of T-bars were used to close the defect. Thoracoscopic repair took the longest time because of trocar placement and dissection of the periesophageal tissue for localization of the defect in the esophagus. Although ECC was the fastest technique, it could not achieve full-thickness repair of the esophageal wall. Moreover, larger gaping defects could not be bridged by the jaws of the clips. In contrast, ECS anchors were deployed across the entire esophageal wall and showed well-healed scares with the smallest remaining gaps. One of the disadvantages of T-bars is that placing them beyond the gastrointestinal wall cannot be performed under direct vision. So, the needle tip may harm or inadvertedly place a T-bar into an unwanted structure as reported in a previous study [30].

The novel over-the-scope clip (OTSC) system showed promising results for gastrostomy closure [31] and has been used in for closure of postoperative leaks following gastrectomy and primary repair after spontaneous acute esophageal perforation [32]. Cardiac septal occluders might be a valuable alternative. Repici et al. have recently reported the first human case of esophagus-tracheal fistula closure by using a cardiac septal occluder with good results [33]. Other prototype suturing/apposition devices might be of future use in esophagotomy closure, namely, Padlock-G clips (Aponos Medical, Kingston, NH, USA) [34], NDO Plicator (NDO Surgical Inc., Mansfield, MA, USA) [35], g-Cath/g-Prox (Usgi Medical Inc, San Clemente, CA, USA) [36], flexible Endostich (Covidien, North Haven, Conneticut, USA) [37], OverStich (Apollo Endosurgery, Austin, TX, USA) [38], Direct Drive Endoscopic System (DDES Boston Scientific, Natick, MA, USA) [39], Anubis-scope (Karl Storz, Tuttlingen, Germany) [40],and Endo-Samurai (Olympus, Tokyo, Japan) [41].

Von Reitein et al. presented a prototype self-expanding metal stent (SX-ELLA stent, ELLA-CS, Hradec Kralove, Czech Republic) for direct incision esophagotomy closure without any suture [22]. Fifteen-millimeter direct incision esophagotomies were created in 12 domestic pigs using a prototype endoscopic Maryland dissector (Ethicon Endosurgery, Cincinnati, OH, USA). Six animals were randomly assigned to open surgical repair and six animals to endoscopic closure using the self-expanding, covered, nitinol stent in a nonsurvival setting. Pressurized leak test results were not different for stent compared to surgical closures. Six animals underwent transesophageal endoscopic mediastinal interventions and survived for 17 days. Stents were extracted at day 10. All survival animals were found to have complete closure and adequate healing of the esophagotomies, without leakage or infectious complications.

Finally, the hybrid approach presented by Rolanda et al. might be useful for safe esophagotomy closure. Using a thoracoscope with a 5 mm working channel, the authors inserted a needle-holder and performed an end-to-end esophageal anastomosis with gastroscopic intruments assistance [18].

## 4. Mediastinum and Pneumothorax Management

Injecting air or carbon dioxide (CO_2_) is a key component for adequate exposure and visualization, especially in thoracic NOTES. Air insufflated in an uncontrolled manner through the endoscope results in wide fluctuations in intrathoracic and intraperitoneal pressures, overdistension of the gastrointestinal tract, and adverse hemodynamic effects. Von Delius et al. studied the potentional cardiopulmonary effects of transesophageal mediastinoscopy in a porcine model, using a conventional gastroscope [42]. Air insufflation was manually performed and the pressure was monitored through the working port of the gastroscope. In 3 of the 8 pigs, there was pleural injury with tension pneumothorax, resulting in hemodynamic instability. In the remaining 5 pigs, median mediastinal pressure maintained was 4.5 mm Hg (mean 5.4 ± 2.2 mm Hg). In this uncomplicated mediastinoscopies, peak inspiratory pressures, pH, partial pressure of CO_2_, and partial pressure of O_2_ were not influenced. 

Inadvertent high-pressure pneumomediastinum and pneumothorax have been major complications since the begining of thoracic NOTES [7, 12, 16]. Most authors use thoracic tube drainage for pressure relief. As CO_2_ pressure control is also a main concern in abdominal endoscopic surgery, new insufflators have been adapted to both deliver and monitor CO_2_ through the endoscope [43]. These may be of some use in transesophageal NOTES. Meanwhile, using a Veress needle or a transthoracic trocar may be a secure way to achieve good pneumothorax pressure control [18].

There is a great debate whether CO_2_ or room air should be used for transesophageal NOTES. CO_2_ is far more soluble in blood than air and fatal CO_2_ embolism is rare. The effect of CO_2_ with respect to laparoscopy has suggested an overall attenuated inflammatory response that may provide a further immunologic benefit. Conversely, room air laparoscopy has been shown to generate a greater inflammatory response, but a recent case-control study did not find a significant difference between the peritoneal inflammatory response of NOTES versus laparoscopy with CO_2_ and air pneumoperitoneum [44].

Even for intraesophageal endoscopic surgery, the question if either air or CO_2_-insufflation should be used is relevant. A study by Uemura et al. found a decreased need for midazolam in patients undergoing esophageal endoscopic submucosal dissection with CO_2_-insufflation when compared to air-insufflation. The authors attributed this decreased need for midazolam to decreased procedural pain [45]. In human POEM procedures, only CO_2_-insufflation has been used [26, 46]. Inoue et al. reported that none of the 17 patients in their series had postoperative subcutaneous emphysema, but CT scan just after procedure revealed a small amount of CO_2_ deposition in the paraesophageal mediastinum. The authors suggest that positive pressure ventilation with intratracheal intubation should be maintained at higher pressures than those generated by endoscopic CO_2_-insufflation in order not only to reduce mediastinal emphysema but also to reduce the risk of air embolization [26].

In their series of 5 patients undergoing POEM, Swaanström et al. observed the development of pneumoperitoneum in 3 patients and placement of a Veress needle was necessary to decompress it [46]. According to the authors, Inoue described this occurrence as well in 10% of this most recent series of more than 100 patients (personal communication) and theorized that it might occur due to gas permeation through the remarkably thin longitudinal muscle fibers of the esophagus [46].

## 5. Infection Prevention

Since the beginning of NOTES procedures, sterility has been a hurdle. Infection must be prevented by using a clean access site. Most transesophageal protocols follow a 12–24-hour liquid formula diet, intravenous antibiotics and esophageal and stomach irrigation with saline or iodopovidone solution. Despite these precautions, even a sterile overtube used to protect the endoscope from oral contamination becomes contaminated on oral insertion and can transport bacteria to the esophagus, the mediastinum, and the thorax.

Several infectious complications have been reported. In a study by Fritscher et al. two out of 12 pigs had reflux of gastric contents into the esophagus that resulted in spillage through the esophagotomy [28]. The study protocol included 12-hour fasting period before surgery and a 3-day antibiotherapy with enrofloxacin. Despite this, one animal died of severe mediastinitis, whereas the other one developed a subclinical mediastinal abscess found on necropsy. The authors suggested that careful aspiration of gastric contents at the beginning of the procedure should always be performed. Also, the authors concluded that 12 hours of fasting may be too short time to clear the stomach of the animals well enough. In a previous study by Gee et al., one out of four animals developed submucosal abscess, despite 24 h liquid diet, esophagus and stomach lavage with iodopovidone solution and cefazolin injection preoperatively [14].

There is also some controversy about the need for endoscope sterilization. In a recent literature review, Spaun et al. concluded that, although difficult, it is possible to terminally sterilize flexible endoscopes. Steris System 1TM that uses 0.2% peracetic acid was the cheapest and fastest sterilization method and scored second in the risk of recontamination. Ethylene oxide gas (ETO) sterilization has the lowest risk of recontamination but is the slowest and most expensive method. The authors recommend sterile instrumentation for clinical NOTES until well-designed and randomized clinical trials are available and guidelines are published [47].

When transferring the results from animal experiments to human settings, one should keep in mind that anatomy and physiology of the esophagus and the mediastinum in humans are somewhat different from those of the pig, especially with regard to wall structure, motility, and infection pathophysiology of the mediastinum. In humans, a perforation of the esophagus causes severe complications or even death in at least 30–50% of cases [48]. In human POEM, patients are placed on a clear liquid diet 24 hours and given a single preoperative dose of a first generation cephalosporin [46]. Although published series account for a short number of patients, no infectious complications were reported. Neither studies specify if the flexible endoscope was either completely sterilized or conventionally disinfected.

## 6. Conclusions

Transesophageal NOTES offers new possibilities in less invasive access to mediastinal and thoracic cavities. Ongoing NOTES revolution permitted the development of esophageal submucosal endoscopic techniques with almost immediate human application. POEM is a perfect example of this. Theoretical advantages of transesophageal NOTES warrant the continuation of research, although some hurdles are to be overcome. The critical nature of the organs that involve the esophagus, the risk of hemodynamic instability related to pressure pneumomediastinum and pneumothorax, and potential infectious complications call for caution when transition to human practice.

**Figure 2:**
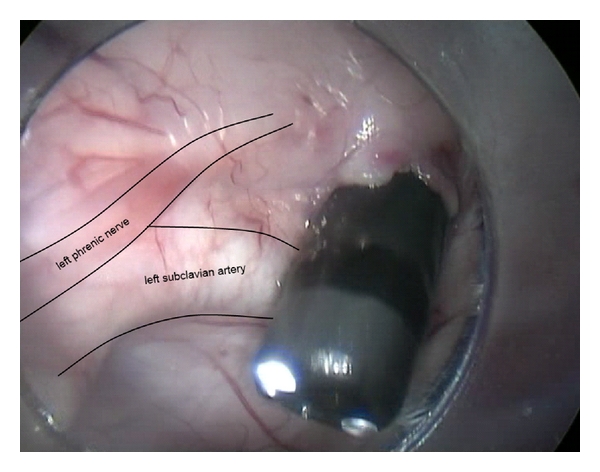
Transthoracic visual control of transesophageal port creation in the upper third of the esophagus (porcine model).

A hybrid NOTES approach, adding transthoracic assistance, might be the key to safe human translation, as it gives visual control of transesophageal port creation (Figure 2), it may improve esophagotomy closure, it permits triangulation and countertraction using flexible instruments inserted through the gastroscope and rigid instruments inserted through the thoracoscope, and it gives a good intrathoracic pressure control and pneumothorax drainage.

## Figures and Tables

**Table 1:** Transesophageal NOTES procedures in animal studies.

Mediastinoscopy	Cardiomyotomy
Thoracoscopy	Esophagomyotomy
Lymphadenectomy	Vagotomy
Pleural biopsy	Sympathectomy
Myocardial and left atrium injection	Esophagectomy and end-to-end anastomosis*
Pericardial fenestration	Pulmonary lobectomy*
Epicardial ablation	Left atrial appendage ligation*

*With single transthoracic trocar assistance.
